# Psychosocial factors associated with overdose subsequent to Illicit Drug use: a systematic review and narrative synthesis

**DOI:** 10.1186/s12954-024-00999-8

**Published:** 2024-04-15

**Authors:** Christopher J. Byrne, Fabio Sani, Donna Thain, Emma H. Fletcher, Amy Malaguti

**Affiliations:** 1 Division of Molecular and Clinical Medicine, School of Medicine, Ninewells Hospital and Medical School, University of Dundee, Dundee, UK; 2 Directorate of Public Health, NHS Tayside, Kings Cross Hospital, Dundee, UK; 3 Division of Psychology, School of Humanities, Social Sciences and Law, University of Dundee, Scrymgeour Building, Dundee, UK; 4 Tayside Drug and Alcohol Recovery Psychology Service, NHS Tayside, Dundee, UK

**Keywords:** Overdose, Psychosocial, Narrative synthesis, Systematic review, Drug-related death

## Abstract

**Background and aims:**

Psychological and social status, and environmental context, may mediate the likelihood of experiencing overdose subsequent to illicit drug use. The aim of this systematic review was to identify and synthesise psychosocial factors associated with overdose among people who use drugs.

**Methods:**

This review was registered on Prospero (CRD42021242495). Systematic record searches were undertaken in databases of peer-reviewed literature (Medline, Embase, PsycINFO, and Cinahl) and grey literature sources (Google Scholar) for work published up to and including 14 February 2023. Reference lists of selected full-text papers were searched for additional records. Studies were eligible if they included people who use drugs with a focus on relationships between psychosocial factors and overdose subsequent to illicit drug use. Results were tabulated and narratively synthesised.

**Results:**

Twenty-six studies were included in the review, with 150,625 participants: of those 3,383–4072 (3%) experienced overdose. Twenty-one (81%) studies were conducted in North America and 23 (89%) reported polydrug use. Psychosocial factors associated with risk of overdose (*n* = 103) were identified and thematically organised into ten groups. These were: income; housing instability; incarceration; traumatic experiences; overdose risk perception and past experience; healthcare experiences; perception of own drug use and injecting skills; injecting setting; conditions with physical environment; and social network traits.

**Conclusions:**

Global rates of overdose continue to increase, and many guidelines recommend psychosocial interventions for dependent drug use. The factors identified here provide useful targets for practitioners to focus on at the individual level, but many identified will require wider policy changes to affect positive change. Future research should seek to develop and trial interventions targeting factors identified, whilst advocacy for key policy reforms to reduce harm must continue.

**Supplementary Information:**

The online version contains supplementary material available at 10.1186/s12954-024-00999-8.

## Introduction

People Who Use Drugs (PWUD) experience myriad harms which drive substantial morbidity and mortality [1–6]. In 2019, approximately 6% of the world’s population used illicit drugs at least once – including using illicitly obtained prescription medications in the context of polydrug use – and this is predicted to rise to 11% by 2030 [7, 8]. Approximately 21% of PWUD are estimated to have experienced recent non-fatal overdose – known to precipitate future fatal overdose – equating to an estimated 3.2 million people, while approximately 42% have ever experienced overdose [2]. Internationally, approximately 500,000-600,000 fatalities are attributable to drug use annually, with close to 80% of these related to opioids and 25–30% directly induced by opioid overdose [7, 9]. This can include illicit drugs, such as heroin, as well as use of illicitly obtained pharmaceutical opioids, such as morphine, fentanyl, and oxycodone [2, 3]. The escalation in drug-related harms and mortality in recent decades has been attributed to a triple-wave epidemic, mediated by supply and demand side drivers, characterised by widespread opioid use; beginning with prescription opioid pills, transitioning through heroin use, and culminating in synthetic opioids – of variable quality and potency – including fentanyl variants, and nitazenes, often combined with or substituted for heroin [10, 11].

In North America alone, nearly 600,000 people have died from an opioid-induced overdose in the last two decades with 1.2 million predicted to meet the same fate by 2029 if current trends persist. Elsewhere in the Americas substantial mortality rates have also been recorded [12, 13]. In the UK and Western Europe, overdose and mortality rates associated with polydrug use are increasing year-on-year in some nations, with opioids involved in most fatalities [14–17]. In Australasia, an estimated 51% of PWUD are reported to have experienced non-fatal overdose, while this is estimated at approximately 34%, 45%, and 50%, in East & Southeast Asia, South Asia, and Central Asia, respectively [2]. Indeed, Asia, relative to North America, Europe, and Australia, has the highest crude mortality rates among PWUD, with many attributable to fatal overdose [3]. Although data from African settings is sparse, the available evidence suggests that overdose consequent to illicit drug use, fatal or non-fatal, is increasingly common worldwide, and constitutes a significant threat to public health. Beyond opioids, other central nervous system depressants – benzodiazepines, alcohol – play a critical role contributing to risk, usually in the context of polydrug use [17]. Similarly, stimulants like cocaine in different forms, and amphetamines, are commonly used together with opioids and elevate risk by artificially masking respiratory depression [17, 18].

Responding to these alarming trends, many have endeavoured to improve surveillance and trial interventions to protect people who use drugs from harm. Some existing medicalised interventions include naloxone provision [19–22], opioid agonist therapy (OAT) [23], opioid antagonist therapy [24], supervised consumption sites [25–27], related healthcare engagement [28], detoxification [29], and integrated prevention activities [30]. Naloxone provision has gained particular salience due to its efficacy in rapidly reversing opioid-induced overdose symptoms [31]. Conventionally carried in medical and pre-hospital settings, evidence has shown high willingness among overdose bystanders to administer it [20, 32, 33]. Subsequently, several countries spanning Europe, Australia, and North America, have adopted legislative changes to enable provision without prescription, and protect bystanders who administer it from prosecution [34–36]. Beyond medicalised interventions, recovery-based approaches which prioritise empowerment, self-determination, and holistic wellbeing, have been widely adopted to underpin recovery journeys with senses of identity, belonging, purpose, and social connection [37]. Peer outreach and in-reach programmes for overdose reduction, as well as mutual help programmes, have also demonstrated efficacious impacts on recovery [38–41]. Such approaches acknowledge that recovery is an ongoing process that requires support, compassion, and dedication, which often extends beyond drug use alone to shifts in identity [42–44].

It is in the context of the varied approaches to overdose intervention, and the acknowledgement that experiences of drug effects are influenced by psychological characteristics and social processes, that we sought to evaluate the available evidence quantifying the risk of overdose among PWUD associated with psychosocial factors [45, 46]. That is, features that pertain to the influence of social factors on an individual’s mind or behaviour, and to the interrelation of behavioural and social factors upon outcomes [47]. These may relate, for example, to social resources, like healthcare access or income source; psychological resources, such as risk perception; and psychological morbidity. Several guidelines on illicit drug use and dependence recommend psychosocial interventions, often targeting behaviour change through mindfulness, motivational interviewing, cognitive behavioural therapy (CBT) based interventions, and acceptance and commitment therapy [48–52]. These interventions are frequently positioned as adjuncts to overall treatment packages, as they are of uncertain benefit relative to medicalised therapies [53–55].

Over the years, many risk factors for overdose have been identified, for example: polydrug use; psychiatric comorbidity; unstable housing; witnessing overdose; substance use disorder; prescription of opioids; increasing pharmacy use; increasing opioid prescribers; vulnerability to socio-economic marginalisation; hepatitis C/HIV infection; male gender; rural residence; certain employment types/industries; incarceration; familial distress; disability; detoxification programme experience; the built environment; and suicidality as key factors [56–67]. However, despite this expansive evidence base, prior to this review, we were unable to identify any unified work that identified which psychosocial factors are associated with overdose, and therefore best to target with interventions found in prevailing guidelines.

Generating this information is critically important in the current era of increasingly limited public health resource and multiple competing public health priorities. Given their prevalence in clinical guidelines, and the uncertainty around their benefits, we sought to understand which psychosocial factors might impact on risk of overdose, to inform future intervention development and clinical practice. Accordingly, we undertook a systematic review with a narrative synthesis, which aimed to identify which, if any, psychosocial factors are associated with risk of overdose, whether fatal or non-fatal.

## Methods

This review complied with the updated PRISMA statement checklist for reporting of systematic reviews and meta-analyses [68] and reporting guidelines for synthesis without meta-analysis in systematic reviews [69]. The review protocol with methods and inclusion criteria was registered in advance on PROSPERO (CRD42021242495).

### Eligibility criteria

Only studies written in English were considered. The search (up to 14 February 2023) was completed with no limitations on publication dates and no geographic restrictions.

### Participants

Studies were required to include PWUD as participants.

### Exposure

The exposure in this study was psychosocial factors which are associated with fatal and non-fatal overdose. Psychosocial was defined as pertaining to the influence of social factors on an individual’s mind or behaviour, and to the interrelation of behavioural and social factors on the outcome [47].

### Comparison

In studies where comparison was undertaken, PWUD who experienced overdose were compared to PWUD who did not.

### Outcome

The primary outcome was overdose (fatal or non-fatal) consequent to use of illicit, or illicitly obtained controlled, drugs. Intentional overdose was excluded where possible, as suicidality constitutes different behavioural characteristics to unintentional overdose. Where it was unclear whether intention was assessed or not, the study was included.

### Design

The review included observational studies (cross-sectional, cohort, case-control, and qualitative studies). Case series, case reports, and reviews, were excluded.

### Information sources

The following databases were searched via OVID: Medline, Embase and PsycINFO. Cinhal was searched via EBSCOhost. Grey literature was explored by searching with Google Scholar. Reference lists of selected full-text studies were manually screened for further identification of relevant studies.

### Search strategy

The search strategy was identical across databases, adjusting for database-specific search requirements. An example of the search strategy is provided in the Supplementary File. Reference lists for manuscripts eligible for full text review were searched manually for relevant titles; whilst Google Scholar was searched with ‘Psychosocial factors AND drug overdose’, and results screened manually. Screening stopped once 100 sequential results did not match search terms, given the results were ordered according to accuracy and relevance. Database searches were saved in an EBSCOhost or OVID account folder. Duplicates were removed.

### Study selection and data extraction

Search results were exported from relevant databases into Microsoft Excel 365 spreadsheets for screening, with tables on study characteristics and psychosocial factors created using Microsoft Word 365. One reviewer (AM) screened titles for inclusion. Two reviewers (AM and CJB) screened all abstracts and full texts independently and a third reviewer (FS) arbitrated. Inter-rater agreement, calculated using Cohen’s kappa in Stata 17 BE, indicated high levels of agreement for both abstract (κ = 0.672 [0.565-0.780], *p* < .001) and full-text (κ = 0.835 [0.697-0.974], *p* < .001) screening. Data were extracted by two reviewers (AM and CJB), and separated into tables. First, data were extracted for study and sample characteristics: author, study design, location and location type, sample size, gender, age, ethnicity, population type, drugs (and other substances) reported, overdose definition, and number who experienced overdose. Second, psychosocial factors associated with overdose identified in each study along with comparators and the estimated effects/description of the association were extracted and tabulated.

### Risk of bias assessment

Two reviewers independently assessed risk of bias for all included studies, discussing any discrepancies and mutually agreeing on final assessment; where required, arbitration was conducted by a third person to arrive at a final decision. The National Institutes of Health Study Quality Assessment Tools for quantitative studies, and the Critical Appraisal Skills Programme Qualitative studies checklist for qualitative studies, were used [70, 71]. In brief, these prompt quality appraisal by considering clarity of research aims; definition of, and homogeneity of, study populations; participation rates; appropriateness of analytic approaches; clarity of outcomes measured; and ethical conduct.

### Effect measures

Effect measures extracted from the studies were tabulated. Given the heterogeneous nature of the studies selected for the review, and the attendant factors examined, results were narratively synthesised; effects were not meta-analysed.

### Synthesis procedure

Data were extracted manually and tabulated according to study characteristics and study findings (identified factor, author, effect size, and direction of effect). The tables were used to familiarise the reviewers with the data initially. Once data extraction was complete, the findings were reviewed, and relationships within the data and overlapping themes were annotated throughout the process of narratively synthesising individual data. The themes were discussed among three members of the research team (AM, CJB, FS) and a peer worker with lived experienced of drug use to ensure they were as accurate a reflection of the lived reality of drug use as could feasibly be achieved for a review. Themes were considered against the review question and full dataset to ensure they were focused and addressed the research question. Extracted data within each theme were then inspected to explore differences in effect direction and potential bias introduced by the different study designs included in the review. Where divergences existed, these were considered in light of study design and risk of bias. Following these steps, the manuscript was drafted, which continued the analytical, procedural, and conceptual thinking for the synthesis to be completed.

## Results

### Study selection

**Fig. 1:**
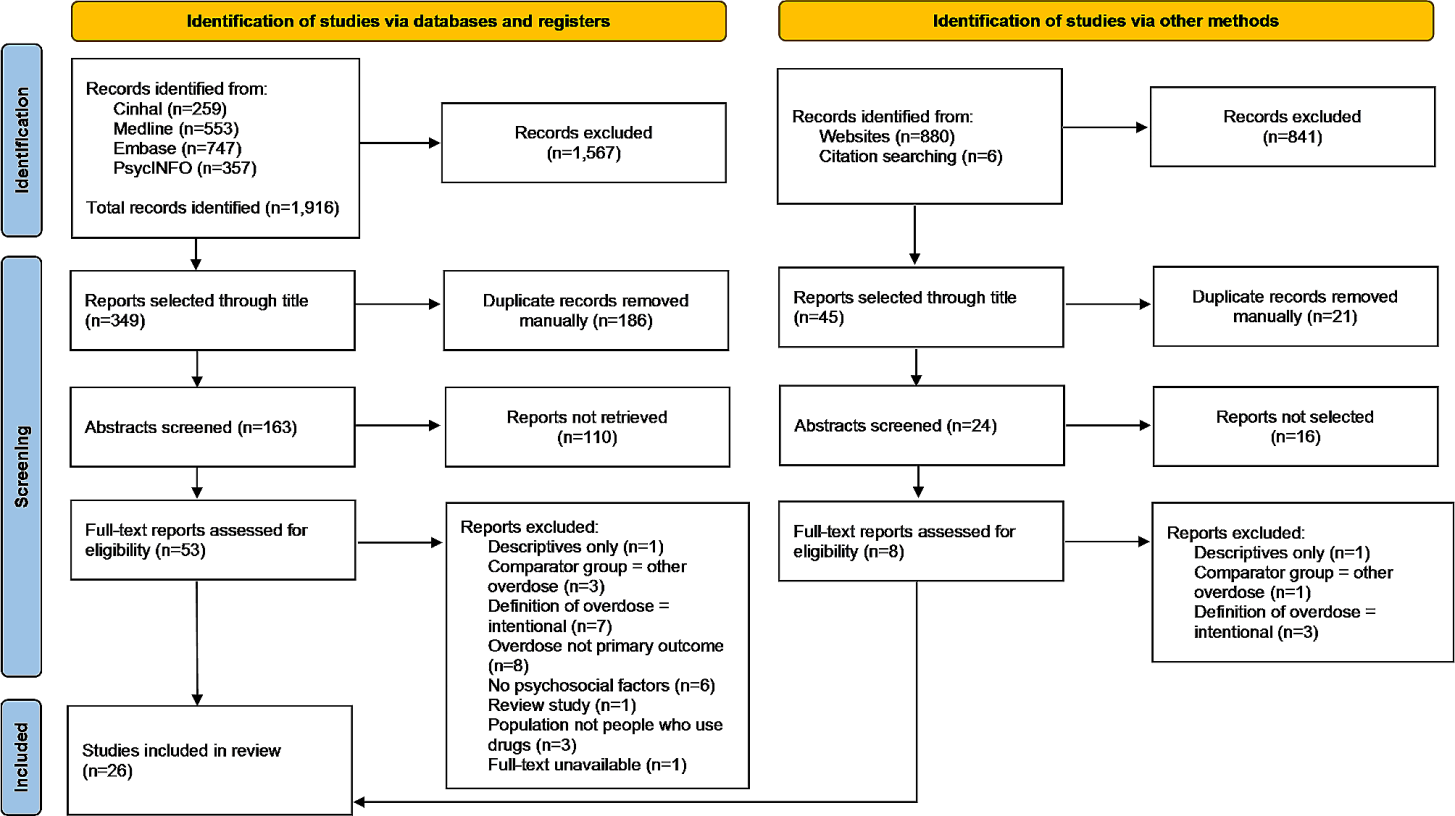
Prisma flow chart summarising the screening process

The screening results are illustrated in Fig. 1. During the search, 2,802 titles were screened: 2,408 were excluded, and 394 were selected for abstract review. After exclusion of duplicates, 187 remained. After further review, 61 were selected for full text assessment. Thirty-five studies were excluded with reason, whilst 26 were selected for quality appraisal and analysis.

### Study characteristics

All studies focussed on overdose, fatal and non-fatal, consequent to illicit drug use as the primary outcome. This was often combined with use of legal substances (e.g. alcohol), and/or illicitly obtained controlled drugs, meaning the cohorts examined were often in the context of polydrug use. One study defined the outcome as death by unintentional overdose, according to post-mortem medical examination records [72], while one examined people hospitalised with ICD-9 codes for opioid-induced non-fatal overdose [73]. All other studies relied on self-reported non-fatal overdose disclosure, though outcome timeframes varied. In nine studies, participants self-reported ever experiencing overdose [74–82]. For nine other studies, the primary outcome was self-reported overdose in the last six months [83–91]. The primary outcome for three studies was experience of overdose in the past 12 months [92–94]. Riggs et al. defined the primary outcome as self-reported overdose in the last three years, while Argento et al. defined it as self-reported overdose during the study observation period (participants were sampled over nine years and follow-up varied) [95, 96]. Lastly, for one study the primary outcome was self-reported overdose in the past five years [97]. Descriptive characteristics of each study are in Table 1.

The total sample comprised 150,625 people. Of those, the number of participants who experienced overdose, according to the definitions reported, ranged from 3,383 to 4,072 (3%). A range is provided as one study did not report the number with sufficient clarity [87].

Most studies were conducted in North America (*n* = 21), three were in Asia, one was in Europe, and one in Australia. Participant ages ranged from 21 to 56 years. Six studies focussed on female and/or gender minority participants [75, 77, 84, 88, 90, 96], and the remainder had a preponderance of male participants (Table 1). Twenty-three studies reported polydrug use and, of those, eight specified this was a mixture of prescription and illicit drugs. Three studies did not disclose the specific drugs used [73, 74, 88].

**Table 1:** Descriptive characteristics of reviewed studies (*n* = 26)

Author	Design	Location (Type)	N	Female	Age^†^	Ethnicity	Population	Substances reported	Primary outcome	N reported OD
Argento et al. 2023	Open prospective cohort	Canada (urban)	273	273 (100%)	*32*	148 (54%) Indigenous 103 (38%) White 22 (8%) Black/ person of colour	Women sex workers	Alcohol Benzodiazepines Cocaine Crystal methamphetamine Opioids	Self-report OD (during follow up)	63 (23%)
Bazazi et al. 2015	Cross-sectional	Malaysia (mixed)	460	17 (4%)	—	416 (90%) Malaysian 44 (10%) Unreported	People who use drugs	Benzodiazepines Buprenorphine Heroin Methamphetamine Methadone	Self-report OD (past 6 months)	92 (20%)
Bonar et al. 2016	Cross-sectional	USA (mixed)	91	21 (23%)	*45*	49 (54%) Non-Caucasian 42 (46%) Caucasian	People who use drugs	—	Self-report OD (ever)	50 (55%)
Chang et al. 2019	Qualitative	USA (mixed)	40	11 (28%)	*43*	25 (63%) White 6 (15%) African American 8 (20%) Hispanic 1 (2%) Bi/multi-racial	People who use drugs	Alcohol Benzodiazepines Cocaine Methamphetamine Opioids	Self-report OD (past 5 years)	40 (100%)
El-Bassel et al. 2020	Cross-sectional analysis of RCT	Kazakhstan (urban)	400	400 (100%)	*34*	—	Women sex workers	Amphetamine Codeine Desomorphine Fentanyl Heroin Methadone Morphine Opioids (prescribed) Opium Sedatives Synthetic opiates Tramadol	Self-report OD (lifetime)/ (past 90 days)	150 (38%)/ 27 (18%)
Fairbairn et al. 2008	Prospective cohort	Canada (mixed)	551	225 (44%)	**39**	186 (36%) Aboriginal 365 (64%) Unreported	People who use drugs	Alcohol Benzodiazepines Crack cocaine Crystal methamphetamine Cocaine Heroin Methadone Morphine Speedball	Self-report OD (past 6 months)	37 (7%)
Goldenberg et al. 2020	Open prospective cohort	Canada (mixed)	624	624 (100%)	**34**	329 (53%) Indigenous 295 (47%) Unreported	Women sex workers	Heroin Crack cocaine Crystal methamphetamine Cocaine Opioids (prescribed)	Self-report OD (past 6 months)	48 (8%)
Grau et al. 2009	Cross-sectional	Russia (urban)	60	29 (38%)	*31*	—	People who use drugs	Alcohol Opioids Amphetamines	Self-report OD (past year)	27 (45%)
Harris et al. 2023	Open prospective cohort	Canada (urban)	857	857 (100%)	**38**	398 (46%) White 458 (54%) Black/ Indigenous/ Person of colour	Women sex workers	Alcohol Opioids Stimulants	Self-reported OD (past 6 months)	305 (36%)
Havens et al. 2011	Cross-sectional analysis of Longitudinal study	USA (rural)	400	165 (41%)	**31**	375 (94%) White 25 (6%) Unreported	People who use drugs	Alcohol Benzodiazepines Cocaine Heroin Hydrocodone Marijuana Methadone Methamphetamine Oxycodone	Self-report OD (ever)	112 (28%)
Lake et al. 2015	Prospective study, retrospective data analysis on baseline.	Canada (urban)	1,697	552 (33%)	**42**	1,044 (62%) Caucasian 651 (38%) Other 2 (0%) Unreported	People who use drugs	Alcohol Cocaine Crack Heroin	Self-reported OD (past 6 months)	372 (22%)
Lamonica et al. 2021	Qualitative	USA (suburban)	32	32 (100%)	*40*	23 (72%) White 6 (19%) African American/ Black 3 (9%) Hispanic	Suburban women	Cannabis Crack cocaine Cocaine Heroin Methamphetamine Opioids (prescribed)	Self-report OD (ever)	32 (100%)
Latkin et al. 2004	Cross-sectional	USA (urban)	742	310 (42%)	*43*	689 (96%) African American 53 (4%) Unreported	People who use drugs	Alcohol Crack cocaine Heroin	Self-report OD (ever)	185 (25%)
Latkin et al. 2019	Clinical trial, but retrospective data analysis on baseline	USA (urban)	444	186 (42%)	**45**	379 (85%) African American 56 (13%) White 9 (2%) Other	People who use drugs	Heroin Opioids (prescribed and illicit) Speedball	Self-reported OD (past year)	166 (37%)
Milloy et al. 2010	Cross-sectional	Thailand (urban)	252	66 (26%)	**37**	—	People who use drugs	Alcohol Benzodiazepines Ecstasy Heroin Methadone Midazolam Yaba	Self-report OD (ever)	75 (30%)
Mitra et al. 2021	Cross-sectional	USA (urban)	48,869	21,433 (44%)	—	35,058 (72%) White 4,694 (10%) Black 1,664 (3%) Hispanic 7,453 (15%) Other	Intensive care admissions	Alcohol Illicit drugs (unspecified)	NFOD by ICD-9 codes: 965.01, E850.0, 965.0, 965.00, 965.02, 965.09, E850.1, E850.2.	171 (0.4%)
Pabayo et al. 2013	Prospective cohort	Canada (urban)	1,931	653 (34%)	—	—	People who use drugs	Alcohol Cocaine Crystal methamphetamine Heroin Methadone Morphine Speed	Self-reported OD (past 6 months)	58–147 (3–8%)
Pizzicato et al. 2018	Retrospective Cohort	USA (mixed)	82,780	66,409 (20%)	**34**	52,954 (64%) Black 15,118 (18%) White 11,965 (15%) Hispanic 2,743 (3%) Other/ Unknown	Prison experienced people	Benzodiazepines Cocaine Fentanyl Heroin	Death by unintentional drug OD	837 (1%)
Riggs et al. 2020	Retrospective cohort	USA (mixed)	5,766	666 (8%)	*56*	2,225 (39%) Black 2,345 (41%) White 1,124 (19%) Other 72 (1%) Unreported	Homeless experienced military veterans.	Alcohol Analgesics Cocaine Fentanyl Gabapentin Heroin Methadone Pregabalin Sedatives	Self-reported OD (past 3 years)	379 (7%)
Schiavon et al. 2018	Prospective cohort	USA (—)	243	105 (43%)	*34*	216 (89%) Caucasian 24 (10%) African American 3 (1%) Unreported	People who use drugs	Heroin Methadone Opioids (prescribed)	Self-reported OD (ever)	107 (44%)
Silva et al. 2013	Cross-sectional	USA (urban)	596	193 (32%)	*21*	333 (56%) White 263 (44%) Non-white	Young people who use drugs (16–25 yrs)	Cocaine Heroin Methamphetamine Opioids (prescribed) Stimulants Tranquilisers (prescribed)	Self-report OD (ever)	138 (24%)
Thumath et al. 2021	Retrospective cohort	Canada (mixed)	696	696 (100%)	**40**	406 (58%) Non-Indigenous 289 (42%) Indigenous	Women sex workers and women living with HIV	—	Self-reported OD (past 6 months)	135 (19%)
Tobin et al. 2007	Longitudinal	USA (urban)	659	292 (44%)	—	632 (96%) African American 27 (4%) Other	People who use drugs	Alcohol Crack cocaine Cocaine Heroin	Self-report OD (ever)	96 (15%)
Tomko et al. 2022	Cross-sectional	USA (urban)	563	222 (40%)	*48*	95 (17%) Non-Hispanic White 397 (72%) Non-Hispanic Black 58 (11%) Hispanic/ Mixed race/ Other race	People who use drugs	Cocaine Fentanyl Heroin Opioids (prescribed) Speedball	Self-reported OD (past 6 months).	168 (30%)
Vallance et al. 2018	Cross-sectional	Canada (urban)	548	171 (32%)	*41*	415 (76%) White/Other 128 (24%) Indigenous	Homeless/ inadequately housed people	Crystal methamphetamine Cocaine powder Crack cocaine Heroin Pharmaceutical opioids	Self-reported OD (past 12 months)	102 (20%)
Winter et al. 2015	RCT	Australia (mixed)	1,051	222 (21%)	—	821 (78%) Unreported 230 (22%) Indigenous	Prison experienced people	Alcohol Amphetamines Cannabis Heroin Opioids (prescribed and illicit)	Self-reported OD (past 6 months)	38 (4%)

**Notes**: Unreported values are denoted with an em dash (—) where relevant. All proportions rounded to the nearest whole number.

**Abbreviations**: OD, overdose; PWID, People Who Inject Drugs; PWUD, People Who Use Drugs.

^†^Age reported is mean or median. Mean denoted with italicised type, median with bold type. Values rounded nearest whole number.

### Methodological quality

No methodological concerns were identified which warranted removal of any of the included studies (Supplementary file 1).

### Psychosocial factors

**Fig. 2:**
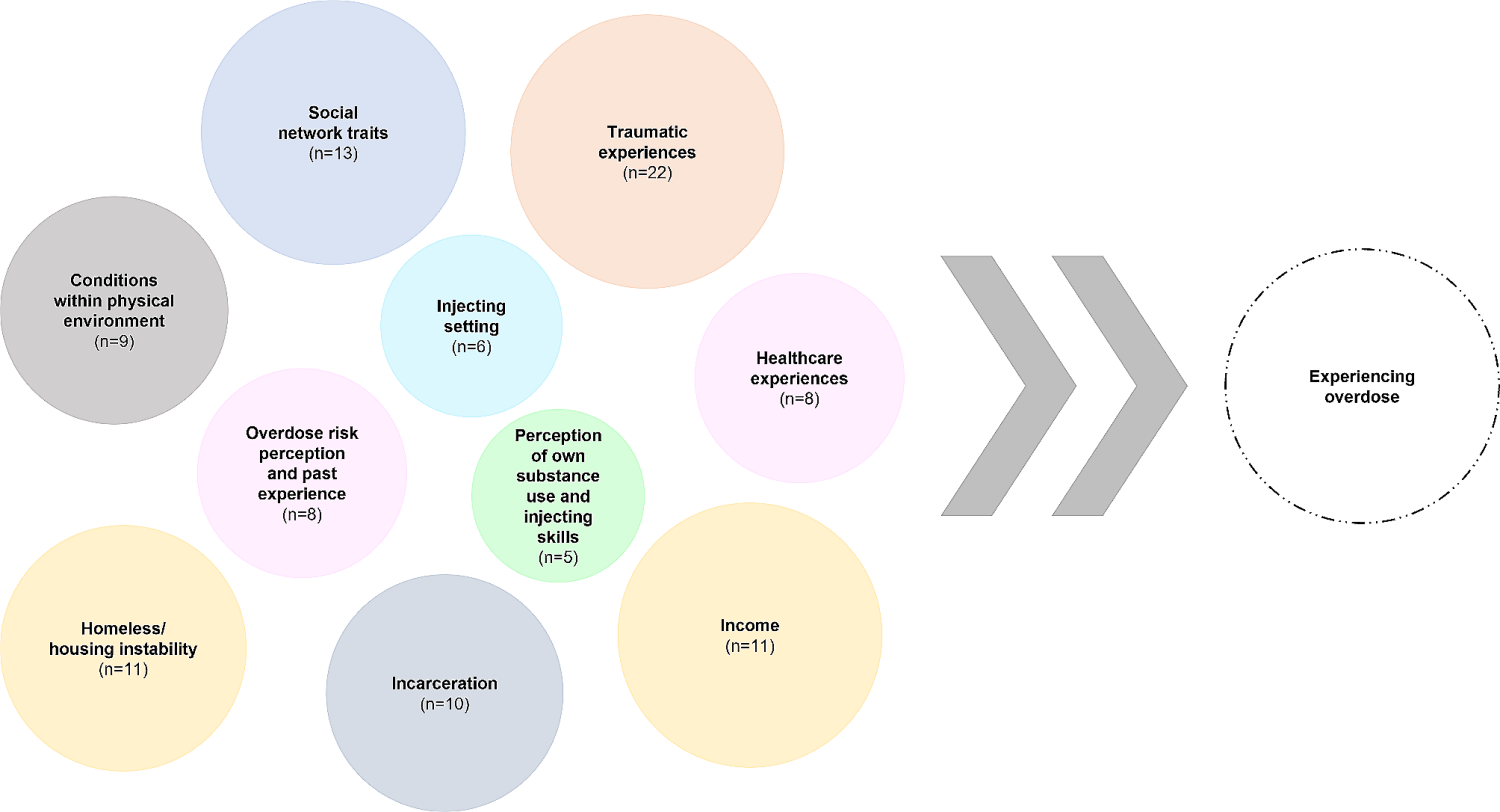
Thematic groups of factors found to impact on experience of overdose in reviewed studies (*n* = 103) **Note**: N in each circle is the number of factors within that thematic group. Groups with smaller N are smaller circles, while groups with the same N are the same colour. Groups are randomly scattered as there is no inherent hierarchy or linearity to their impact

Factors associated with overdose (*n* = 103) were extracted from each study and structured into ten thematically similar groupings (Table 2; Fig. 2).

Eighteen studies reported odds ratios (OR) as the measure of the association between factors and exposure to overdose [73, 75, 78–88, 90, 91, 93–95]. Two studies reported incidence rate ratios (IRR) [74, 76], two reported relative risk (RR) [89, 92], and two reported hazard ratios (HR) [72, 96]. Two studies were qualitative, so no quantitative estimates were reported [77, 97]. Given the heterogeneity of measures and study designs, summary statistics were not calculated, and meta-analysis was not performed [98]. Despite this heterogeneity, estimates of effects were considered and informed the narrative synthesis.

### Income

Eight studies explored the relationship between income source and/or unemployment and odds, or risk, of overdose [73, 75, 81, 85, 87, 89, 90, 94]. Winter et al. demonstrated sustained unemployment prior to imprisonment was associated with four-to-five times higher risk of overdose following liberation. Mitra et al. also showed a four-fold increase in odds associated with unemployment. Similarly, Pabayo et al. found 40% and 70% higher odds of overdose among men and women respectively, in receipt of social welfare. Harris et al. showed recent engagement in sex work was associated with 60% higher odds of overdose, while Fairbairn et al. reported ever engaging in sex work was associated with twice the odds. El-Bassel et al. examined compounding effects of sex work and violence, with over ten years sex work experience also associated with twice the odds of overdose, and combined exposure to this with recent violence, including from intimate partners, increasing the odds four-fold. Analysis from Latkin et al. (2019) implied selling drugs in the past 30 days was associated with two-to-three times higher odds of overdose. Finally, work by Silva et al. found identifying as a lower socio-economic status growing up increased odds of overdose by 80%.

### Homeless/housing instability

Eight studies explored this theme [73, 81, 87–91, 95]. Unstable housing and lack of accommodation was consistently found to increase the odds and risk of overdose. Mitra et al. observed the largest effect, with housing insecurity increasing the odds of overdose seven-to-eight-fold. Thumath et al. found recent homelessness was associated with 60% higher odds, current homelessness increased odds by 30% according to Riggs et al., while being unhoused in the past six months was associated with 50–70% increased odds in a study by Harris et al. in an all-female sample, and 30% higher odds in Pabayo et al. in a restricted male-only analysis. The highest estimate among examinations of recent homelessness was by Silva *et* al, who showed past 90-day homelessness increased odds of overdose by close to three-fold, while Tomko et al. estimated a two-fold increase. Ever experiencing homelessness and ever living in a foster home were associated with five-fold and 60% increases in odds of overdose in work by Thumath et al. and Silva et al. respectively. Finally, Winter et al. found experience of unstable accommodation one month prior to incarceration increased risk of overdose three-fold among recently liberated prisoners.

### Incarceration

Eight studies explored incarceration-related factors [72, 75, 77, 79, 81, 86, 89]. Winter et al. estimated any previous incarceration as an adult resulted in five-times higher risk of overdose, while Milloy et al. and El-Bassel et al. estimated a roughly four-fold increase in odds of overdose for participants with similar histories, and Silva et al. estimated a doubling of odds. Harris et al. and Lake et al. found incarceration in the past six months was also associated with twice the odds of overdose, with the effect enduring when adjusted for physical or emotional neglect in the work by Lake et al. El-Bassel et al. estimated a more pronounced effect among those with history of incarceration and intimate partner violence, who experienced five-times higher odds of overdose, with those who experienced non-partner violence having close to four-times higher odds. Recent liberation from prison, coupled with mental ill health, conferred a 50% higher hazard of overdose in work by Pizzicato et al. and Lamonica et al., in their qualitative study, also found that recent liberation from carceral settings increased risk of overdose in a suburban all-female cohort.

### Traumatic experiences

Nine studies assessed traumatic experiences [75, 77, 84, 86, 88–91, 96]. Lamonica et al. found emotional trauma, such as negative life events and consequent depressive states, increased risk of overdose. Various other traumatic experiences were examined, but multiple iterations of physical trauma pre-dominated. Thumath et al. found experience of intimate partner violence doubled the odds of overdose among marginalised women in Canada, Lake et al. found physical abuse and neglect increased odds of overdose by 40% and 30% respectively. Harris et al. found recent physical violence increased overdose odds by 80% in an all-female cohort, with that increasing to close to three-fold among sex workers and adjusted for confounders. Combined physical and sexual workplace violence was associated with twice the odds of overdose among sex workers in Goldenberg et al., while sexual abuse carried a 50% increase in odds in Lake et al., and any physical/sexual violence conferred a 90% increase in hazard in Argento et al. El-Bassel et al. examined multiple type of physical violence, imparted by intimate partners and others, and found consistently elevated odds of overdose, with severe physical violence conferring 30% increased odds in adjusted analysis.

Beyond physical trauma, Tomko et al. identified a 70% increase in odds of overdose among those who experience daily psychological pain in adjusted analysis. Separately, severe emotional abuse conferred a 50% increase in odds in adjusted analysis by Lake et al. Adverse childhood events, such as removal from family as a child, or removal from parental care, were associated with a four-fold increase in odds by Winter et al. and a doubling of odds by Thumath et al., respectively. Similarly, having a child removed from one’s care held a 60% increase in odds in adjusted analysis by Thumath et al., and child custody loss was linked with higher overdose risk in qualitative work by Lamonica et al. Finally, Thumath et al. found food insecurity drove a 90% increased in odds of overdose.

### Overdose risk perception and past experience

Risk perception and past experiences with overdose were evaluated in six studies [74, 77, 80, 81, 92, 95]. There were divergent effects between perceived severity of prior overdose experience and participants’ perception of their own susceptibility to overdosing in work by Bonar et al., where higher perceived severity was linked to 40% decreased incidence and higher perceived susceptibility was linked to 50% higher incidence. Vicarious experience, i.e. witnessing an overdose, was associated with two-fold higher odds of subsequent overdose experience in Riggs et al., while ever witnessing a family member overdose conferred 60% higher odds in adjusted analysis by Silva et al. Schiavon et al. estimated that the higher the number of times a participant witnessed another person overdose, odds of subsequent overdose experience increased by 40%, with odds increasing four-fold where the other person was identified as a friend. Prior experience of overdose was also linked to 70% higher risk of subsequent overdose in Grau et al. whereas, in qualitative work by Lamonica et al., being a ‘novice’ to drug use, which may include erroneous polydrug use, was linked to higher risk.

### Healthcare experiences

Most healthcare experiences, across eight studies, focused on medicalised addictions treatment [76, 80, 81, 84, 86, 89, 91, 94]. Ever experiencing addictions treatment was associated with a 60% increased incidence of overdose in Havens et al., while Latkin et al. estimated a 50% increase in odds. However, when examined by Silva et al., the increase in odds was two-fold, and ever receiving opioid substitution therapy conferred a three-fold increase in relative risk in Winter et al. Schiavon et al. estimated that with increasing number of treatment episodes, the odds of experiencing overdose increased by 60% in adjusted analysis. Conversely, Lake et al. found that being denied access to addictions treatment was associated with close to three-fold odds of overdose. Other studies examined healthcare need, with Goldenberg et al. identifying unmet healthcare need was associated with 70% higher odds of overdose, and Tomko et al. linking unmet mental health care need to a 40% increase in adjusted analysis.

### Perception of own drug use and injecting skills

Three studies examined participants’ perceptions of their own drug use, two of which were qualitative [77, 95, 97]. In the quantitative work, Riggs et al. estimated that participants who perceived they had a drug ‘problem’ had five-fold higher odds of subsequent overdose in adjusted analysis. Lamonica et al. found participants who disclosed a lack of knowledge about drug use, a lack of control over the quality of the drugs they were using, or lack of knowledge of their tolerance of those drugs, had higher risk of experiencing overdose. Chang et al. termed similar types of knowledge as ‘opioid expertise’ – this also included perceived self-control over opioid use and one’s bodily response – and identified that participants who felt they possessed a high degree of opioid expertise had increased risk of overdose. Related to the sense of expertise and experience, low injecting skill was examined in two studies [86, 87]. Both linked requiring assistance with injecting with increased odds of overdose. Lake et al. found requiring help to inject increased odds by 90%, with adjusted models for physical and sexual abuse yielding 70% higher odds, and adjusted models for physical and emotional neglect yielding 70% and 50% higher odds respectively. Likewise, Pabayo et al., found that, among men, requiring help injecting increased odds of overdose by 74%.

### Injecting setting

Injecting setting was assessed in four studies [83–86]. Injecting in public spaces in the past six months was consistently linked with higher odds of overdose. Lake et al. found a close to three-fold increase in odds of overdose in a Canadian cohort, which attenuated to 90% when adjusted for experience of emotional abuse, and to 70% when adjusted for experience of emotional neglect. Fairbairn et al. estimated a more pronounced effect, with a close to five-fold increase in odds associated with injecting in public settings. Both cohorts were sampled in Vancouver, Canada. Conversely, these studies found diverging effects for injecting alone in the last six months. Lake et al. estimated an 80% increase in odds, while Fairbairn et al. found the odds of overdose decreased by 60%. Fear of police intervention while injecting in public spaces was associated with a two-fold increase in odds by Bazazi et al., including in adjusted analysis. While ‘rushed’ outdoor drug use in the last six months conferred a 30% increase in odds in work by Goldenberg et al.

### Conditions within physical environment

In related analyses, specific conditions within the wider physical environment were found to mediate overdose likelihood in six studies that examined this [83, 84, 90, 93, 94, 96]. Proximity to harm reduction provision was examined in three studies, with somewhat diverging outcomes. First, Bazazi et al., found that among those who reported that a needle and syringe provision (NSP) site was the main source of their injecting equipment acquisition, this was linked to a 60% reduction in odds of overdose. However, Latkin et al. (2019) found that among those who replaced syringes through such a service, there was a three-to-four-fold increase in odds. Vallance et al. also reported a similar finding, where participants that resided in areas of high harm reduction coverage had twice the odds of overdose in adjusted analysis. In further conflicting results, Goldenberg et al. identified police-related barriers to harm reduction access doubled odds of overdose in adjusted analysis.

Similarly, Argento et al., found the same parameter conferred a close to three-fold increase in hazard of overdose in adjusted analysis, while Harris et al. observed that, among women, being stopped, searched, detained, or assaulted by police conferred a 50% increase in odds. This increased to a doubling of odds when stratified for sex workers only. Meanwhile, living in an area characterised by criminalisation, marginalisation, and prevalence of drug use, was associated with 40% higher odds of overdose in the same paper. Somewhat similar to wider drug use prevalence in the area, residing in a neighbourhood with an increasing number of known settings in which to use drugs was associated with 30% increase in odds overdose in adjusted analysis by Latkin et al.

### Social network traits

Finally, density of social networks and supports were examined in six studies [76–78, 82, 84, 87]. Pabayo et al. found three or more social supports was associated with a 50% reduction in odds of overdose among women in adjusted analysis. While, in their study, Tobin et al. found density of social network at baseline, and increases in density reported during follow-up, were associated with 90% and 80% reductions in odds in adjusted analyses. However, among those who reported recent injection drug use, Tobin et al. found increasing density in social network conferred a 20% increase in overdose odds in adjusted analysis, while Latkin et al. (2004) identified that reporting increasing numbers of people who inject heroin in one’s social network was associated with 20% higher odds of past overdose, and 30% higher odds of recent overdose. Conversely, in the same study, increasing numbers of contacts who snort heroin, rather than inject, was associated with a 20% reduction in odds of overdose.

Conflicting somewhat with these findings, Tobin et al. also found that, among those who reported recent injection drug use, an increasing number of people who inject drugs in participants’ social networks was associated with 80% reduced odds of overdose in adjusted analysis. Similarly, Havens et al. found increasing numbers of support members in one’s social network was linked to a 20% increased in incidence of overdose in adjusted analysis. Latkin et al. found increasing levels of conflict within a participant’s social network conferred a 30% increase in odds, whilst other studies examined intimate partnerships. In their qualitative study, Lamonica et al., found being friends, or in an intimate partnership, with someone who uses drugs increased participants’ risk of overdose. Similarly, Goldenberg et al., reported that providing drugs for an intimate partner (who was male) was associated with a 40% increase in odds of overdose.

**Table 2:** Factors associated with overdose categorised by theme

Theme	Referent	Factor	Paper	Estimate	Adjusted estimate
**Income**	No	Sex work (last six months)	Fairbairn	OR: 2.1 (1.0-4.3)	…
	No	Sold Drugs (past 30 days).	Latkin (2019)	OD < 1 year ago: OR: 2.7 (1.1–6.6)	…
	< 10 years	> 10 years sex work, ever OD.	El-Bassel	…	aOR: 2.5 (1.5–4.3)
	< 10 years	> 10 years sex work, recent Intimate partner violence, recent OD.	El-Bassel	…	aOR: 4.1 (1.4–11.8)
	< 10 years	> 10 years sex work, recent non partner violence, recent OD.	El-Bassel	…	aOR: 4.0 (1.4–11.6)
	No	Unemployed 6 + months prior to prison	Winter	RR: 5.4 (2.2–12.9)	aRR: 4.4 (1.9–10.4)
	No	Welfare receipt (males only).	Pabayo	…	aOR: 1.4 (1.1-2.0)
	No	Welfare receipt (females only).	Pabayo	…	aOR: 1.7 (1.2–2.4)
	Middle/upper class	Lower social class growing up.	Silva	OR: 1.8 (1.3–2.7)	aOR: 1.8 (1.2–2.8)
	No	Sex work (last six months)	Harris	OR: 1.6 (1.3-2.0)	…
	No	Unemployed.	Mitra	OR: 4.3 (2.7-7.0)	…
**Homeless/** **housing instability**	No	Past 90-day homelessness.	Silva	OR: 2.7 (1.7–4.1)	…
	No	Homeless last six months (males only).	Pabayo	…	aOR: 1.3 (1.0-1.6)
	No	Current homelessness.	Riggs	…	aOR: 1.3 (1.0-1.7)
	No	Ever homeless.	Thumath	OR: 4.9 (2.3–10.5)	…
	No	Recent homeless (last six months).	Thumath	OR: 1.6 (1.1–2.3)	…
	No	Unstable accommodation in month prior to imprisonment.	Winter	RR: 2.8 (1.0-7.4)	…
	No	Ever lived in foster home.	Silva	OR: 1.6 (1.0-2.3)	…
	No	Unhoused last six months.	Harris	OR: 1.7 (1.4–2.1)	…
	No	Unhoused last six months (sex workers only).	Harris	OR: 1.5 (1.1–2.1)	…
	No	Housing insecurity.	Mitra	OR: 7.5 (3.6–13.8)	…
	No	Recent homelessness (last six months).	Tomko	OR: 2.1 (1.5–2.9)	aOR: 1.9 (1.3–2.7)
**Incarceration**	No	Incarceration (past 6 months).	Lake	OR: 2.2 (1.5–3.3)	With physical neglect: aOR: 2.3 (1.8–2.8) With emotional neglect: aOR: 1.9 (1.5–2.4)
	No	Ever incarcerated.	Milloy	OR: 4.4 (1.8–10.8)	aOR: 3.8 (1.5–9.7)
	N/A	Having recently been released from drug treatment or incarceration.	Lamonica	QS (increased risk)	QS (increased risk)
	No	History of incarceration.	El-Bassel	…	aOR: 4.3 (2.6–7.3)
	No	History of incarceration with recent intimate partner violence.	El-Bassel	…	aOR: 4.7 (1.8-12.48)
	No	History of incarceration with recent non partner violence.	El-Bassel	…	aOR: 3.68 (1.4–9.5)
	No	History of previous adult incarceration.	Winter	RR: 4.9 (1.4–16.8)	…
	No	Ever incarcerated.	Silva	OR: 2.2 (1.4–3.5)	…
	No	Serious mental illness (among recently liberated)	Pizzicato	…	aHR 1.5 (1.3–1.9)
	No	Incarceration (last six months).	Harris	OR: 1.9 (1.5–2.6)	…
**Traumatic experiences**	N/A	Emotional trauma (negative life events, states of depression)	Lamonica	QS (increased risk)	QS (increased risk)
	No	Food insecurity (last six months)	Thumath	OR: 1.9 (1.3–2.8)	…
	No	Child removed (ever).	Thumath	OR: 1.8 (1.2–2.7)	aOR: 1.6 (1.0-2.4)
	N/A	Child custody loss.	Lamonica	QS (increased risk)	QS (increased risk)
	No	Removed from family as a child.	Winter	RR: 5.8 (2.4–13.9)	aRR: 4.4 (1.8–10.9)
	No	Removal from parental care.	Thumath	OR: 2.3 (1.5–3.4)	…
	None/low	Moderate/severe emotional abuse.	Lake	…	aOR: 1.5 (1.2–1.9)
	No	Intimate Partner Violence (last six months).	Thumath	OR: 2.2 (1.2–3.9)	…
	No	Severe physical violence (ever).	El-Bassel	OR: 1.6 (1.1–1.6)	aOR: 1.3 (1.0-1.6)
	No	Multiple types of violence (ever).	El-Bassel	OR: 1.8 (1.4–2.3)	…
	No	Recent severe physical violence recent coupled with recent intimate partner violence.	El-Bassel	OR: 1.4 (1.00-1.9)	…
	No	Multiple types of recent violence coupled with recent intimate partner violence.	El-Bassel	OR: 1.5 (1.00-2.1)	…
	No	Recent severe physical violence coupled with recent non- partner violence.	El-Bassel	OR: 1.4 (1.1–1.7)	aOR: 1.3 (1.0-1.7)
	No	Multiple types of recent violence coupled with recent non- partner violence.	El-Bassel	OR: 1.7 (1.2–2.5)	…
	None/low	Moderate/severe physical abuse.	Lake	OR: 1.5 (1.0-2.2)	aOR: 1.4 (1.1–1.7)
	None/low	Moderate/severe sexual abuse.	Lake	OR: 1.6 (1.1–2.3)	aOR: 1.5 (1.2–1.9)
	None/low	Moderate/severe physical neglect.	Lake	…	aOR: 1.3 (1.0-1.6)
	No	Physical and/or sexual workplace violence (last six months).	Goldenberg	OR: 2.1 (1.5–2.9)	…
		Any physical/sexual violence.	Argento	HR: 1.9 (1.1–3.2)	…
	No	Violence last six months.	Harris	OR: 1.8 (1.5–2.2)	…
	No	Violence last six months (sex workers only).	Harris	OR: 2.2 (1.6–2.9)	aOR: 2.6 (1.9–3.5)
	Never	Daily psychological pain.	Tomko	OR: 2.4 (1.7–3.3)	aOR: 1.7 (1.1–2.5)
**Overdose risk perception and past experience**	Five-point scale	Overdose perceived severity (increasing).	Bonar	IRR: 0.6 (0.4–0.8)	…
	Five-point scale	Overdose perceived susceptibility (increasing).	Bonar	IRR: 1.5 (1.0-2.8)	…
	N/A	Low understanding of overdose risk (novice to drug use, polydrug use).	Lamonica	QS (increased risk)	QS (increased risk)
	Increasing number	Previous overdose.	Grau	…	aRR: 1.7 (1.1–2.6)
	No	Witnessed overdose.	Riggs	…	aOR: 2.0 (1.6–2.4)
	Increasing number	Number of times seen others overdose.	Schiavon	…	aOR: 1.4 (1.1–1.8)
	Increasing number	Past friend overdose.	Schiavon	…	aOR: 4.21 (2.0-8.9)
	No	Ever witnessed family member overdose.	Silva	OR: 2.1 (1.4–3.1)	aOR: 1.6 (1.0-2.5)
**Healthcare experiences**	No	Unmet need for health services (last six months).	Goldenberg	OR: 1.7 (1.2–2.3)	…
	No	Ever in addictions treatment.	Havens	IRR: 2.1 (1.3–3.4)	aIRR: 1.6 (1.0-2.5)
	No	Denied access to addictions treatment (past 6 months).	Lake	OR: 2.5 (1.4–4.6)	…
	No addictions treatment	Any addictions treatment.	Latkin (2019)	OD > 1 year ago: OR: 1.5 (1.1-2.0)	…
	No	Ever on opioid substitution treatment.	Winter	…	aRR: 3.0 (1.2–7.6)
	No	Ever in addictions treatment.	Silva	OR: 2.4 (1.7–3.6)	…
	Increasing number	Number of treatment episodes at buprenorphine clinic.	Schiavon	…	aOR: 1.6 (1.2–2.1)
	No	Unmet mental health need.	Tomko	OR: 1.9 (1.4–2.4)	aOR: 1.4 (1.0-1.9)
**Perception** **of own drug use and injecting** **skill**	No	Self-reported drug problem (last 12 months)	Riggs	…	aOR: 5.1 (4.1–6.4)
	N/A	Lack of knowledge or control (drug quality) of tolerance.	Lamonica	QS (increased risk)	QS (increased risk)
	N/A	Sense of ‘opioid expertise’ (experience, knowledge, tolerance, and self-control regarding opioids)	Chang	QS (increased risk)	QS (increased risk)
	No	Required help injecting (past 6 months).	Lake	OR: 1.9 (1.3–2.7)	With physical abuse: aOR: 1.7 (1.4–2.1) With sexual abuse: aOR: 1.7 (1.4–2.1) With physical neglect: aOR: 1.7 (1.4–2.1) With emotional neglect: aOR: 1.5 (1.2–1.9)
	No	Required help injecting (males only).	Pabayo	…	aOR: 1.74 (1.1–1.8)
**Injecting setting**	No	Injecting alone (last six months).	Lake	OR: 1.8 (1.1–2.9)	…
	No injecting alone.	Injecting alone (last six months).	Fairbairn	OR: 0.4 (0.2–0.8)	…
	No public injecting.	Public injecting (last six months).	Fairbairn	OR: 4.7 (2.4–9.4)	…
	No	Rushed injection from fear of police (last six months)	Bazazi	OR: 2.0 (1.3–3.3)	aOR: 1.9 (1.1–3.6)
	No	Rushed outdoor drug use (last six months)	Goldenberg	OR: 1.3 (1.0-1.6)	…
	No	Injecting in public (last six months).	Lake	OR: 2.8 (1.9–4.1)	With emotional abuse: aOR: 1.9 (1.6–2.4) With emotional neglect: aOR: 1.7 (1.3–2.1)
**Conditions within physical environment**	No	Police-related barriers to harm reduction (last six months).	Goldenberg	OR: 1.7 (1.3–2.2)	aOR: 2.2 (1.6–2.9)
	No	Needle exchange main source of injecting equipment (last six months).	Bazazi	OR: 0.4 (0.2–0.6)	…
	No	Replaced syringes through exchange service (last six months).	Latkin (2019)	OD < 1 year ago: OR: 3.7 (1.8–8.1) OD > 1 year ago: OR: 3.3 (2.1–5.2)	…
	Area with less coverage	Resident in area with high harm reduction coverage.	Vallance	OR: 2.2 (1.4–3.4)	aOR: 2.2 (1.3–3.8)
	Fewer settings	Number of drug use settings (per additional setting) [last six months]	Latkin (2019)	OD < 1 year ago: OR: 1.5 (1.2–1.8) OD > 1 year ago: OR: 1.3 (1.1–1.6)	OD < 1 year ago: aOR: 1.3 (1.0-1.7) OD > 1 year ago: aOR: 1.3 (1.1–1.6)
	No	Police-related barriers to harm reduction (last six months)	Argento	HR 3.0 (1.8–4.9)	aHR 2.6 (1.5–4.5)
	No	Stopped, searched, detained, or assaulted by the police.	Harris	OR: 1.5 (1.1–2.2)	…
	No	Lives in area characterized by prevalent drug use, marginalization, and criminalization.	Harris	OR: 1.4 (1.1–1.7)	…
	No	Stopped, searched, detained, or assaulted by the police (sex workers only).	Harris	OR: 1.6 (1.1–2.3)	aOR: 2.0 (1.3-3.0)
**Social network traits**	Increasing number	Number of support members in social network.	Havens	IRR: 1.3 (1.1–1.6)	aIRR: 1.2 (1.0-1.4)
	None	Three or more social supports (females only).	Pabayo	…	aOR: 0.5 (0.3–0.9)
	Increasing number	Density of social network at visit 1.	Tobin	…	aOR: 0.1 (0.0-0.4)
	Increasing number	Change in density of social network over study.	Tobin	…	aOR: 0.2 (0.1–0.5)
	Increasing number	Density of social network at visit 1 (recent injectors).	Tobin	…	aOR: 0.1 (0.0-0.5)
	Increasing number	Change in density of social network over study (recent injectors).	Tobin	…	aOR: 1.2 (1.1–1.4)
	Increasing number	Number of social network members participant had conflict with.	Latkin (2004)	OR: 1.3 (1.1–1.5)	…
	N/A	Being with friends/partners that use drugs.	Lamonica	QS (increased risk)	QS (increased risk)
	No	Provide drugs for intimate male partner (last six months).	Goldenberg	OR: 1.4 (1.0-1.9)	…
	Increasing number	Number of injectors in social network over study (recent injectors only).	Tobin	…	aOR: 0.2 (0.1–0.5)
	Increasing number	Number in social network who snort heroin.	Latkin (2004)	OR: 0.8 (0.7–0.9)	…
	Increasing number	Number in social network who inject heroin (past overdose).	Latkin (2004)	OR: 1.2 (1.0-1.4)	…
	Increasing number	Number in social network who inject heroin (recent overdose).	Latkin (2004)	OR: 1.3 (1.0-1.5)	…

**Abbreviations**: OR, odds ratio; aOR, adjusted odds ratio; IRR, incidence rate ratio; OD, overdose; RR, relative risk; aRR, adjusted relative risk; aHR, adjusted hazard ratio; N/A, not applicable; QS, qualitative study; …: not modelled or not statistically significant in specific analysis.

Note: All quantitative estimates are rounded to nearest whole number and one decimal place, due to heterogeneity in reporting accuracy across studies

## Discussion

This review is the first to our knowledge which specifically evaluated psychosocial factors associated with unintentional overdose consequent to illicit drug use, with many reviewed studies documenting polydrug use. Prior research suggests the majority of serious overdoses are unintentional, implying our findings are pertinent to the experiences of many people who use drugs [99]. While existing review evidence has elucidated many important factors, as noted in the Introduction, none highlighted the important connections between sex work, violence, or social networks, and overdose risk that we identified [56–67]. Twenty-six studies from seven countries were reviewed, only two of which were qualitative, with the vast majority conducted in North America. Most participants were male, though several studies examined female-only cohorts. The overall proportion estimated to have experienced overdose was 3%, contrasting sharply with global estimates of 21% (15-26%) of PWUD reported to have recently experienced overdose [2]. Sample sizes varied widely, with two registry studies reporting disproportionately large samples relative to other reviewed studies, and low relative overdose prevalence [72, 73]. Excluding these from the estimate would bring the overall prevalence closer to 16%. Thus we believe most studies reviewed are representative of the at-risk population.

Identified factors were structured into ten overarching groups, with some thematically similar correlates yielding conflicting results. Factors varied from the individual (e.g. risk perception) to the structural (e.g. housing) in a manner which illustrates the synergies between biological factors, psychological traits, and social processes, both at micro and macro levels, which influence an individual’s likelihood of experiencing overdose [45, 46, 100, 101].

For example, income played an important role in mediating risk, with experience of sex work, unemployment, drug selling, social welfare receipt, and lower socio-economic status, all associated with increased reports of overdose. The relationship between income and health may be explained by subjective psychosocial experiences mediated by work environments and exposure to unemployment [102, 103]. However, the correlates reported are characterised by socioeconomic marginalisation, which speaks to the economic and political frameworks which worsen health outcomes for people who use drugs within the model of interdiction which predominates globally. For instance, at the micro level, while the individual acts involved in drug use may have shaped sex worker/client interactions and were important in moderating overdose risk, the ultimate harm induced by that behaviour was enabled by the fact sex workers were reticent to report overdose due to criminalisation and structural stigmatisation, both of their drug use but also their method of income generation [104]. The risk environment for sex workers was elucidated further by El-Bassel et al. who demonstrated the compounding impact of violence and sex work on overdose risk [75]. The context may then be at least partially characterised by risky drug use and frequent violence at the micro level – a common experience among sex workers operating in a social environment of gendered norms and unequal power dynamics – which is enabled by public policy at the macro level which marginalises sex workers and leaves them vulnerable to harms related to drug use [105, 106]. These findings speak to the urgent need to cease using criminal law to enforce morals upon income generation and strengthen the previously elucidated case for this as the best strategy to reduce harms experienced by sex workers [107].

At the individual level, there is little evidence to support the use of psychosocial interventions to improve health and well-being among sex workers, perhaps due to the structural factors at play [108]. Separate to this, unemployment was generally associated with higher risk than sex work and other income factors such as social welfare receipt, participation in the illicit drug trade, and lower socio-economic status, and it is important to note that the relationship between these factors and overdose may be mediated by social capital and isolation [59, 62, 109]. These, in turn, drive worse psychosocial outcomes, which are enabled by prevailing policies of state-imposed methods of control (social welfare) of non-conforming behaviour (non-participation in ‘normative’ modes of economic activity), and intentional criminalisation of drug use which erodes drug supply quality and increases overdose risk [10].

In a similar vein, housing instability was consistently linked with increased odds of overdose, similar to prior research which observed this [110]. Among vulnerable adults experiencing homelessness, psychological and social issues at the micro level, such as self-esteem, social support, coping mechanisms, and emotional distress, have been associated with increased substance use [111]. Further, people facing homelessness experience frequent stigmatisation which negatively impacts mental health and well-being, and wider social interactions. Whilst drug use in this context of unstable housing will be influenced by immediate social norms of the situation, there is an overarching synergy between housing and drug use which has driven opioid-overdose to be a leading cause of death among people experiencing it [112–114]. Research suggests this synergy confers 38% higher odds of overdose [115]. These issues are likely manifestations of both immediate social interactions in the context of insecure housing, and macro housing policy which inhibits the social environments which vulnerable individuals are enabled to access. Recent work has reported positive effects for psychosocial interventions in reducing psychological morbidity among people experiencing homelessness [116], but these will not negate the risks which require wider policy reform around housing programmes [112]. For example, many housing programmes restrict PWUD accessing their services as a matter of policy, despite housing being linked with harm reduction impacts and improved psychosocial measures which may facilitate recovery-based approaches [117–119]. The results illustrate a need for supportive and stable housing – a fundamental requirement to establish a sense of safety and stability – to be viewed as a critical intervention which policy makers and public health practitioners should seek to deliver to moderate prevalence of overdose.

The likelihood of becoming homeless may be mediated by history of incarceration [120]. Incarceration was consistently linked to higher risk of overdose in reviewed studies, and other work not reviewed here [115]. The circumstances surrounding the first two weeks post liberation have been demonstrated to induce an up to eight-fold increase in risk of fatal overdose relative to subsequent weeks and, furthermore, all-cause mortality is up to 12.7 times higher than that of the general population among those recently liberated, with most attributable to fatal overdose [121, 122]. While mental health difficulties, victimisation, and feeling unsafe during incarceration, have been linked to poorer psychosocial adjustment upon liberation (which psychosocial interventions may help address), these findings emphasise the inadequacy of efforts by health and welfare services, and carceral establishments, to assist people in the vulnerable period following liberation with transitional social and medical supports [123–125].

Research has shown relapse to drug use in this window occurs in the context of poor social support, situational stressors (violence, poverty, isolation, availability), and decreased tolerance [125]. Conversely, exposure to factors which address these, such as housing, social supports (including avoiding old social networks), mutual help programmes, and spiritual services, have been cited as protective [125]. Overdose risk caused by liberation to environments that trigger drug use may be somewhat ameliorated by provision of take-home naloxone, but research has shown people in prison may not be receptive to training and carriage of naloxone, and motivation to carry it is complicated by desires to remain abstinent [126, 127]. Beyond individual factors, useful conceptual frameworks have been posited to frame the multilevel nature of the determinants involved in overdose risk upon liberation, which suggest researchers shift the lens through which this issue framed from the individual to the socio-structural [128, 129]. Our findings highlight the harms conferred by structural control mechanisms which reinforce criminalisation of drug use and compound inequalities experienced by people who use drugs in health outcomes.

There were additive effects for incarceration with physical neglect and recent experience of violence. Intimate partner violence (IPV) was among the traumatic experiences linked to higher risk, alongside multiple types of intimate partner and non-partner violence, including sexual abuse and neglect. It was unclear from the results whether IPV, abuse, and neglect experienced were reciprocal/bidirectional, however all but one study examining these experiences were in female cohorts. So the relationship between overdose risk and these factors may be understood as the confluence of the drug effects, the norms and boundaries concerning gender-based violence within the immediate social context, and wider cultural and systemic factors which perpetuate gender-based violence. At the individual level, psychosocial interventions, with advocacy and psychological components, can reduce depressive symptomology and post-traumatic stress among IPV survivors, which may ameliorate overdose risk [130]. However, they do not mitigate against re-experience and therefore policy changes which address the physical, social, and economic circumstances that manifest in the macro environment, and perpetuate gender-based violence, are critical to reducing risk, alongside individual interventions. One relevant example is the ongoing pilot of discreet payments to women availing of aid services in Scotland to abscond from circumstances of abuse [131].

In studies which examined experiences of healthcare, unmet needs and denied care were important in elevating overdose risk. PWUD are less likely to be able to avail of preventive healthcare to screen and manage conditions due to frequent experiences of stigma, distrust, and frustration in health environments; with those same people often blamed for the stigma they experience [132–136]. Unmet health needs have been linked to increased depression, with 29% (21-37%) of PWUD meeting the threshold for clinical depression diagnosis, and consequent self-harm and post-traumatic stress common [1, 137]. There were also associations between experience of addictions treatment and overdose which were unexpected, given OAT is known to be protective against drug-related mortality [138]. This association may be explained by severity of dependence (and related suboptimal dosage); changes in tolerance whilst engaging with treatment; those who engaged with treatment having a higher likelihood of follow-up for overdose; those with past overdose experience being more likely to be referred for treatment; OAT discontinuity and re-entry; and transferring between OAT providers [139, 140]. It should further be acknowledged that, though it is an established harm reduction tool, OAT can (and has) been interpreted as a mechanism of control through which moral discipline is inculcated in people who participate in drug use [141, 142]. Through this lens, OAT engagement is necessitated only by ongoing interdiction and the intersecting inequalities and harms this produces. Safer supply and decriminalisation of drug use present reasonable (structural) approaches relative to individual interventions such as OAT, which may aid in mitigating overdose risk at the population level, whilst simultaneously mitigating against negative effects of interventions premised on ill-conceived moral frameworks [143, 144].

Some environmental factors linked to overdose included experience of police-related interventions such as blocking access to harm reduction, stopping, arresting, and detaining people. All of which are more likely to occur in areas characterised by socio-economic marginalisation and prevalent drug use. Policing of drug use is characterised by violence which drives increased psychological distress among PWUD [145, 146]. Similarly, rushed and public injecting, often accompanied by punitive policing, drove increased risk, as demonstrated in previous work [115]. Social-ecological frameworks have been proposed to articulate a means of addressing such factors, as it is unlikely individual-level interventions will modify these risks [147, 148]. It is likely public health approaches which account for the societal, communal, and interpersonal factors, which drive these risks will be required to mitigate against the high likelihood of overdose they confer. These approaches require policy change – particularly regarding criminalisation of drug use and associated policing – while educational campaigns and clear service pathways to harm reduction are also critical.

At a more individual level, perception and social issues noted highlight the interconnectedness between drug use, individual psychology, and social processes. Social support systems impact psychological and physical wellbeing, and the interplay of social networks with environmental and individual factors can differentially impact upon psychological stressors [149]. This was apparent in the results, with contrasting effects observed. Higher density of social networks of varying degrees were protective against overdose in one study [82], while others which examined social networks characterised by conflict, ongoing injecting, and exposure to recent overdose among peers, signalled harmful impacts. Individually, peer social support may reduce psychological distress which in turn reduces overdose risk [150, 151], and interventions which target social connectedness may be beneficial in this context [152]. More broadly, these results may be viewed through the Social Identity Model of Recovery, which proposes that recovery from drug use relies on a shift in identity wherein individuals reshape their social network to one wherein drug use is uncommon [43, 44, 153]. Reviewed studies which signalled harmful impacts studied social networks characterised by ongoing risks, whilst one might infer that those which examined network density where actually examining surrogates of networks wherein use of drugs was less prevalent. Where recovery from drug use is sought, peer support can be critical. One form which this takes is in mutual aid groups, which have been shown to catalyse changes in social networks, increase recovery capital, and enhance commitment to sobriety, through community reinforcement [154, 155]. Additionally, alternative unstructured peer support strategies, such as recovery cafes, can also be enabling, whilst strategies like ‘spotting’ can help to enhance overdose response in the context of ongoing drug use [156, 157].

Furthering the consideration of social context, witnessing overdose is deleterious to psychological wellbeing, causes post-traumatic stress, and can drive people to engage in risky drug use behaviours to manage feelings of bereavement and trauma [158, 159]. Psychological distress has itself been independently associated with close to ten-times higher odds of overdose in young people [110]. Therefore trauma-informed psychosocial interventions for post-traumatic stress – which have been demonstrated as effective, particularly CBT-based therapies – may be important to integrate into existing harm reduction services [160, 161]. Particularly when prefaced by safety and stabilisation work within a phased interventional model, to establish safety and create coping mechanisms before trauma reprocessing occurs [162]. However, an increase in psychological wellbeing may not mitigate against social factors such as requiring injecting assistance – shown previously to increase risk by approximately 58% – and risk conferred by one’s perception of their drug use [115]. Factors which implied low injecting skill were associated with increased risk – psychosocial interventions may improve injecting skills among PWUD [163] – alongside identifying as an expert in drug use. This contrasts with research among people who use new psychoactive substances, where expertise has been linked to higher risk perception and greater control in exposure to risk [164]. Individual-level interventions which assess and affect changes to psychological mechanisms that relate risk perception to overdose risk may therefore also be appropriate to explore.

### Limitations

There are several limitations to this review. First, we did not undertake a meta-analysis due to the heterogeneity in effect estimates and study designs, instead opting for narrative review of the effects. Although appropriate for the heterogeneous study types and factors examined, this provides limited information for decision making relative to meta-analysis and risks emphasising the results of some studies erroneously and potentially misrepresenting the evidence [165]. Second, reviewed studies were concentrated in high-income countries, mostly in North America, significantly limiting the generalisability of the work. No work from African settings was identified, which is a critical limitation given the ongoing epidemic of extra-medical use of opioids (tramadol) and expansion of cocaine markets in recent years into African and Near and Middle Eastern settings, beyond conventional markets in Europe and North America [166]. This likely means PWUD in these settings will be disproportionately impacted by associated harms in coming years, with little representation in research. Third, our search strategy included terms for ‘psychosocial’, ‘psychological’, ‘social’, or ‘behavioural’, which was intended to be comprehensive. Nonetheless, some relevant research may have been omitted unintentionally due to the search design and/or interpretation of the results by the reviewers, given the broad scope and interpretability of the term ‘psychosocial’; we mitigated against this by referencing a recognised definition when interpreting and extracting results, and citing works thought to be relevant in the Discussion [47]. Finally, only two studies reviewed were qualitative in nature. This suggests the findings may omit relevant work documenting subjective experience, not captured in the quantitative studies. We suggest two reasons for this: our search strategy did not include terms for methodology like ‘quantitative’ or ‘qualitative’ which may have resulted in more results returned for relevant qualitative work; and much qualitative work proximal to overdose which we reviewed for inclusion concurrently examined factors which made them ineligible on the basis of our criteria (e.g. suicidal ideation; relationships).

## Conclusions

Globally, rates of fatal and non-fatal overdose continue to increase, alongside many cognate harms, consequent to illicit drug use [1, 2, 167]. This review identified many psychosocial correlates of overdose which spoke to the interdependencies between drug use, psychological traits, and social processes, alongside the overlapping structural, societal, and environmental inequities which govern harms related to drug use, and therefore frame the risks related to overdose. Existing harm reduction interventions are insufficient to resolve the crisis of overdose and avoidable fatalities consequent to the opioid epidemic [168]. To date, many national drug policies are premised more on ideology than evidence, and our findings support the view that punitive approaches are not just ineffective in reducing prevalence of overdose, but actually contribute to the risk environment which increases it [144]. Where we believe this review adds value for the harm reduction movement is in elucidating several themes not previously identified in existing review evidence, which may be helpful in policy work concerning drug use, and clarifying the factors which practitioners may seek to engage at the individual level when exploring psychosocial interventions in harm reduction services, to facilitate therapeutic response. For example: mechanisms underlying risk perception, social connectedness, coping mechanisms, and screening and management of IPV [50–52, 55].

### Electronic supplementary material

Below is the link to the electronic supplementary material.

Supplementary Material 1

## Data Availability

As this was a systematic review, no original data was generated in completing the work. All cited works are listed in the References for consultation.
