# Supplementary material for: Psychosocial factors associated with overdose subsequent to Illicit Drug use: a systematic review and narrative synthesis
Source: Harm Reduct J. 2024 Apr 15;21:81. doi: 10.1186/s12954-024-00999-8 (PMC11017611; doi:10.1186/s12954-024-00999-8)
Supplement: Supplementary file 1 — Supplementary Material 1 [file 12954_2024_999_MOESM1_ESM.docx]

**SUPPLEMENTARY FILE 1**

**Title**

Psychosocial Factors associated with Overdose Subsequent to Illicit Substance Use: A systematic review and narrative synthesis.

**Authors**

Christopher J Byrne^1,2^ Fabio Sani^3^ Donna Thain^2^ Emma H Fletcher^2^ Amy Malaguti^3,4^

**Affiliations**

^1^Division of Molecular and Clinical Medicine, School of Medicine, Ninewells Hospital and Medical School, University of Dundee, Dundee, UK

^2^Directorate of Public Health, NHS Tayside, Kings Cross Hospital, Dundee, UK.

^3^Division of Psychology, Scrymgeour Building, School of Social Sciences, University of Dundee, Dundee, UK

^4^Tayside Drug and Alcohol Recovery Psychology Service, NHS Tayside, Dundee, UK

| **Supplementary Table 1:** CINHAL (via EBSCO) literature search. | |
| --- | --- |
| **Search ID** | **Search** |
| S1 | (MH "Overdose") OR "overdose" |
| S2 | TI ( drug* OR substance* ) OR AB ( drug* OR substance* ) |
| S3 | TI ( (abuse* OR addict* OR dependen* OR use* OR misus* OR disorder* OR intoxicat* OR inject*) ) OR AB ( (abuse* OR addict* OR dependen* OR use* OR misus* OR disorder* OR intoxicat* OR inject*) ) |
| S4 | S2 AND S3 |
| S5 | TI ( (IDU* OR PWID* OR PWUD* OR people who use drugs OR people who inject drugs) ) OR AB ( (IDU* OR PWID* OR PWUD* OR people who use drugs OR people who inject drugs) ) |
| S6 | S1 OR S4 OR S5 |
| S7 | TI ( (psychological OR social OR psychosocial OR behavio$ral) ) OR AB ( (psychological OR social OR psychosocial OR behavio$ral) ) |
| S8 | TI ( (risk OR associat* OR relat*) ) OR AB ( (risk OR associat* OR relat*) ) |
| S9 | TI ( (predict* OR factor* OR variable* OR correlat*) ) OR AB ( (predict* OR factor* OR variable* OR correlat*) ) |
| S10 | S8 AND S9 |
| S11 | S7 AND S10 |
| S12 | TI ( Drug overdose OR overdose* OR fatal overdose* OR non-fatal overdose* OR non fatal overdose OR poisoning OR drug death* OR drug-related death* OR drug related death* OR DRD OR NFOD) ) NOT TI ( suicide OR intention* ) |
| S13 | AB ( Drug overdose OR overdose* OR fatal overdose* OR non-fatal overdose* OR non fatal overdose OR poisoning OR drug death* OR drug-related death* OR drug related death* OR DRD OR NFOD) ) NOT AB ( suicide OR intention* ) |
| S14 | S12 OR S13 |
| S15 | S6 AND S11 AND S14 |

| **Supplementary Table 2: quality appraisal results.** | | | | | | | | | | | | | | | | | | | | | | | |
| --- | --- | --- | --- | --- | --- | --- | --- | --- | --- | --- | --- | --- | --- | --- | --- | --- | --- | --- | --- | --- | --- | --- | --- |
| **Cross-sectional studies** | | | | | | | | | | | | | | | | | | | | | | | |
| **Paper** |  | | **Q1** | | **Q2** | | **Q3** | | **Q4** | | **Q5** | | **Q6** | | **Q7** | **Q8** | **Q9** | **Q10** | **Q11** | **Q12** | **Q13** | **Q14** | **Rating** |
| Bazazi | 2015 | | Y | | Y | | Y | | Y | | Y | | N | | N | Y | N | N | Y | NA | NA | Y | Good |
| Bonar | 2016 | | Y | | Y | | Y | | Y | | N | | N | | Y | Y | Y | N | Y | NA | NA | Y | Fair |
| El-Bassel | 2020 | | Y | | Y | | Y | | Y | | Y | | N | | N | Y | Y | N | Y | NA | NA | Y | Good |
| Grau | 2009 | | Y | | Y | | Y | | Y | | Y | | N | | N | Y | Y | N | Y | NA | NA | Y | Good |
| Havens | 2011 | | y | | y | | y | | y | | y | | n | | n | y | Y | N | Y | NA | NA | Y | Good |
| Latkin | 2004 | | Y | | Y | | Y | | Y | | Y | | N | | N | Y | Y | N | Y | NA | NA | Y | Good |
| Milloy | 2010 | | Y | | Y | | Y | | Y | | Y | | N | | N | Y | Y | N | Y | NA | NA | Y | Good |
| Mitra | 2021 | | Y | | Y | | CD | | Y | | Y | | N | | N | CD | N | N | Y | N | NA | Y | Fair |
| Silva | 2013 | | Y | | Y | | Y | | Y | | Y | | N | | Y | Y | Y | N | Y | NA | NA | Y | Good |
| Tomko | 2022 | | Y | | Y | | CD | | Y | | Y | | N | | N | Y | Y | N | Y | N | NA | Y | Fair |
| Vallance | 2018 | | Y | | Y | | Y | | Y | | Y | | N | | N | Y | Y | N | Y | NA | NA | Y | Good |
| **Cohort studies** | | | | | | | | | | | | | | | | | | | | | | | |
| Argento | | 2023 | | Y | | Y | | CD | | Y | | Y | | Y | Y | Y | Y | N | Y | NA | NA | Y | Good |
| Fairnbairn | | 2008 | | Y | | Y | | Y | | Y | | Y | | N | Y | Y | Y | N | Y | CD | NA | Y | Good |
| Harris | | 2023 | | Y | | Y | | CD | | Y | | Y | | Y | Y | Y | Y | N | Y | N | Y | Y | Good |
| Lake | | 2015 | | Y | | Y | | Y | | Y | | Y | | Y | N | Y | Y | N | Y | NA | NR | Y | Good |
| Pabayo | | 2013 | | Y | | Y | | Y | | Y | | Y | | Y | Y | Y | Y | Y | Y | NA | NR | Y | Good |
| Pizzicato | | 2018 | | Y | | Y | | Y | | Y | | Y | | Y | Y | Y | Y | Y | Y | NA | NA | Y | Good |
| Riggs | | 2020 | | Y | | Y | | N | | Y | | Y | | Y | Y | Y | Y | N | Y | NA | NA | Y | Good |
| Schiavon | | 2018 | | Y | | Y | | Y | | Y | | Y | | N | N | Y | Y | N | Y | CD | NA | Y | Good |
| Thumath | | 2021 | | Y | | Y | | Y | | Y | | Y | | N | Y | Y | Y | N | Y | CD | NR | Y | Good |
| **Clinical trial with retrospective analysis** | | | | | | | | | | | | | | | | | | | | | | | |
| Latkin | | 2019 | | Y | | Y | | Y | | Y | | Y | | N | Y | Y | Y | N | Y | CD | NR | Y | Good |
| **RCT** | | | | | | | | | | | | | | | | | | | | | | | |
| Winter | | 2015 | | Y | | CD | | Y | | N | | Y | | Y | Y | NR | NR | CD | N | N | Y | NR | Good |
| **Longitudinal** | | | | | | | | | | | | | | | | | | | | | | | |
| Goldenberg | | 2020 | | Y | | Y | | Y | | Y | | Y | | Y | Y | N | CD | N | N | NR | Good | | |
| Tobin | | 2007 | | Y | | Y | | Y | | Y | | Y | | Y | Y | NA | Y | N | N | N | Good | | |
| **Qualitative** | | | | | | | | | | | | | | | | | | | | | | | |
| **Paper** | |  | | **Q1** | | **Q2** | | **Q3** | | **Q4** | | **Q5** | | **Q6** | **Q7** | **Q8** | **Q9** | **Q10** | | **Rating** | | | |
| Chang | | 2019 | | Y | | Y | | Y | | Y | | Y | | N | CD | Y | Y | Good value | | Good | | | |
| Lamonica | | 2021 | | Y | | Y | | Y | | Y | | Y | | N | N | Y | Y | Good value | | Good | | | |
| **Abbreviations:** Q*^n^*, question*^n^*; Y, yes; N, no; CD, cannot determine; NA, not applicable. | | | | | | | | | | | | | | | | | | | | | | | |
